# Prevalence of overweight and malnutrition among ethnic minority children and adolescents in China, 1991–2010

**DOI:** 10.1038/srep37491

**Published:** 2016-11-24

**Authors:** Sifan Guo, Chunhua Zhao, Qinghua Ma, Hong-peng Sun, Chen-wei Pan

**Affiliations:** 1 Department of Child Health, Jiangsu Key Laboratory of Preventive and Translational Medicine for Geriatric Diseases, School of Public Health, Soochow University, Suzhou, 215123, P.R. China; 2 The 3rd People’s Hospital of Xiangcheng District, Suzhou, 215134, P.R. China

## Abstract

This study aimed to determine the trends in prevalence of childhood overweight and malnutrition in a large Chinese ethnic minority population from 1991 to 2010. In the Chinese National Survey on Students’ Constitution and Health from 1991 to 2010, multistage stratified sampling was conducted in the series of cross-sectional studies. Participants were 7–18-year-old students randomly selected by sex and region, and included Han and 26 ethnic minorities. During the survey period, the overall prevalence of overweight increased from 5.8% to 13.5%, and malnutrition trend increased from 3.6% to 4.1% in ethnic minority children and adolescents. Moreover, Korean and Mongol children were more likely than Han children to be obese (Korean: RR = 1.52; 95% CI: 1.48–1.56; Mongol: RR = 1.24; 95% CI: 1.20–1.28). Among these minorities, the Dongxiang and Li children were more likely to be malnourished (Li: RR = 1.47; 95% CI: 1.37–1.57; Dongxiang: RR = 1.45; 95% CI: 1.34–1.58). Shui, Khalkhas, Lisu, and Monguor children were less likely to be overweight and malnourished compared with the Hans. The prevalence of overweight among ethnicities increased yearly while that for malnutrition has fluctuated over the past few decades.

Overweight and malnutrition are major health concerns in many low- and middle-income countries, including China^1^,^2^. Overweight children and adolescents are more likely to have metabolic abnormalities including increased blood cholesterol, triacylglyceride and glucose levels, insulin resistance, and hypertension^3^,^4^. Obesity in childhood may lead to a range of adult chronic diseases and conditions, such as cardiovascular diseases, diabetes, and premature death^5^. Furthermore, an unhealthy figure caused by overweight or dysplasia by malnutrition adversely influences children’s mental health^6^,^7^. Conversely, malnutrition among children and adolescents could affect their physical growth and physical work capacity^8^. Particularly, severe malnutrition in the teenage years could delay or cause problems with the development of secondary sex characters^9^. Malnutrition is also an underlying risk factor for morbidity and mortality of many diseases in both children and adults^5^. Childhood malnutrition has been reportedly associated with socioeconomic disadvantages, such as low adult income, poor economic growth, and intergenerational transmission of poverty^10^. Therefore, health-care expenditure to solve or alleviate overweight and malnutrition in children and adolescents is always a heavy financial burden nationally.

Many studies have reported the prevalence of overweight and obesity among children and adolescents in China, but those on malnutrition remain sparse. Concerns are increasing regarding epidemic trends in overweight and obesity, especially among the Hans. However, the prevalence rates of overweight and malnutrition among minorities remain unknown, with the exception of some studies on Tibetan, Mongolian and Uygur children^11^,^12^,^13^. The overall population of 55 ethnic minorities has exceeded 100,000,000 people, accounting for 8% of China’s entire population. Examining both overweight and malnutrition among ethnic minorities using nationally representative samples allows us to recognize growth and development problems experienced by children and adolescents in China.

Thus, this study aims to describe the prevalence rates of overweight and malnutrition among school-aged children and adolescents from different ethnic groups.

## Methods

### Data sources and sampling frame

The Chinese National Surveys on Students’ Constitution and Health (CNSSCH) is a series of complex multistage, cross-sectional, nationwide surveys on the physical fitness and health status of students in China. Becoming a continuous survey in 1985, the CNSSCH has released data every 5 years since, with currently six completed surveys.

In this study, Han participants were school students aged 7–18 years, randomly selected from 31 mainland provinces. The participants were classified by gender and region (urban or rural) within each province, and divided into four groups with equal sample sizes from three socioeconomic classes (upper, middle, and lower). The prevalence of overweight among Han children and adolescents has been reported elsewhere^14^.

Participants of ethnic minorities were primary and secondary school students aged 7–18 years, randomly selected from 13 provinces. Mongolian, Hui, Uyghur, Zhuang and Korean student participants were classified by gender and region (urban and rural). Other ethnic minorities were classified by gender only, not by region (Supplementary Figure 1), and were therefore categorized into other areas. We define rural areas as scattered settlements consisting of labourers engaged in agricultural production. Urban areas are defined as settlements that give priority to non-agricultural economy, including cities and towns. Other areas (other ethnic minorities) are ethnic enclaves where ethnic minorities live together and cannot be categorized into urban or rural areas. This report is based on survey data collected in 1991 (N = 55451), 1995 (N = 54276), 2000 (N = 58551), 2005 (N = 73122), and 2010 (N = 80082).

Our study was approved by the ethics committee of the Medical College of Soochow University in Suzhou, Jiangsu, China, and followed the tenets of the Declaration of Helsinki. Verbal informed consent was obtained from all students aged 7–18 years and from their parents after the nature of the study had been explained.

### Clinical measurements

Data were obtained from normal students who were well-developed, physically and psychologically healthy, and able to participate in various sports activities. Students who had multiple organ dysfunction (such as heart, liver, spleen and kidney), physical disability and deformation, acute diseases (such as high fever and diarrhoea without recovery of physical strength), and females who were menstruating were excluded from participating in the fitness test of the survey. Height (cm) and weight (kg) were measured by the same trained technicians. Weight was measured to the nearest 0.1 kg using a balance-beam scale while the participants were wearing lightweight clothing. Height was measured to the nearest of 0.1 cm with a portable stadiometer while the participants were barefoot (Supplementary Tables 2, 3, 4 and 5). Weight categories among children and adolescents were based on standardized weight-for-height criteria from the sex-and-age-specific 80^th^ percentile weight to the same height population. The standards were created from 1985 CNSSCH data. Overweight was defined as >110% of the standard weight-for-height, after adjusting for age and sex^15^. Malnutrition was defined as <80% of the standard weight-for-height.

### Data analysis

We estimated distributions of overweight and malnutrition by gender and residence (urban, rural, or other area). P < 0.05 was considered statistically significant. To test for trends in the prevalence rates of overweight and malnutrition among children and adolescents during 1991–2010, we used generalized linear models (GLM) with a log function based on binomial distribution. The GLM was also used to generate rate ratios (RR, i.e., prevalence ratio and risk ratio) between the Han and ethnic minority groups, after adjusting for age, group, sex, and region. The overall prevalence rate of ethnic minority groups was normalized by means of weighting by the population of ethnicities from China’s 2010 census data. Age-adjusted prevalence of every ethnic minority was also directly adjusted according to China’s 2010 census population age structure. Data were analysed using SAS (Version 9.1; SAS Institute Inc., Cary, NC., USA) statistical software.

## Results

### Overall trend for overweight and malnutrition

From 1991 to 2010, the overall prevalence of overweight significantly increased among ethnic minority children and adolescents from 5.8% to 13.5% (RR, 1.24; 95% CI, 1.23–1.25) (Table 1). However, the overall malnutrition prevalence increased in 1995 but decreased in 2005 (Table 2). Although overweight and malnutrition affected fewer male participants than female, a higher prevalence of overweight was found in males than females since 2005.

**Figure 1:**
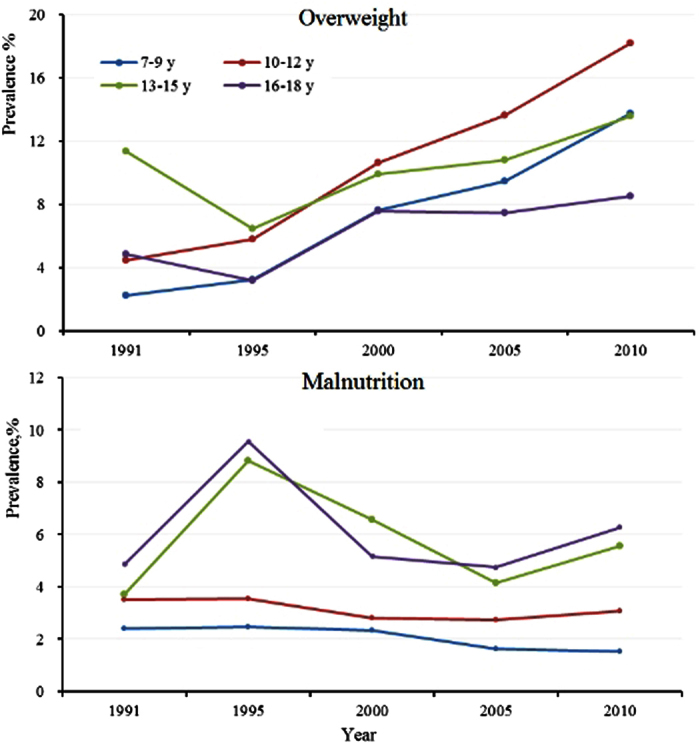
Prevalence of overweight/malnutrition among minority groups by age group from 1991 to 2010. Note: The prevalence rates were adjusted according to the minority population based on the sixth nationwide population census (2010).

A lower rate of prevalence for overweight and malnutrition was found among children living in rural regions compared with those living in urban regions (overweight: RR, 0.84; 95% CI, 0.81–0.87; malnutrition: RR, 0.88; 95% CI, 0.87–0.90). Likewise, a lower rate of prevalence in other regions was found when compared with that in urban regions (overweight: RR, 0.64; 95% CI, 0.62–0.65; malnutrition: RR, 0.88; 95% CI, 0.86–0.91) (Table 3). The prevalence of overweight increased in all age groups from 1991 to 2010, among which the 10–12-year age group had the highest overweight prevalence (RR, 1.40; 95% CI, 1.36–1.44). However, in these same years, the prevalence of malnutrition remained stable and the highest prevalence of malnutrition occurred in the 13–15-year age group (RR, 3.30; 95% CI, 3.21–3.29) (Fig. 1 and Table 3).

### Ethnic Characteristics

**Figure 2:**
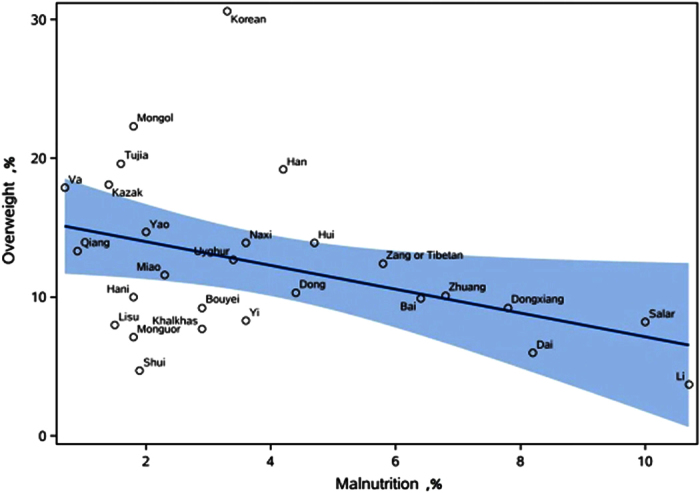
Prevalence of overweight and malnutrition among Chinese aged 7–18 years in 2010.

In 2010, the Koreans had the highest overall prevalence of overweight at 30.6% (95% CI, 29.4–31.9%), followed by the Mongolians at 22.3% (95% CI, 21.0–23.7%) (Table 4). Among ethnic minorities, the Lis had the lowest overweight prevalence (3.7%) and the highest prevalence of malnutrition (10.7%). The Shui, Khalkhas, Lisu, and Monguor children were found to have a low prevalence for overweight and malnutrition (Table 4 and Fig. 2). The Li (10.7%), Salar (10.0%), and Dai children (8.2%) ranked in the top three minority groups for overall malnutrition prevalence. Meanwhile, the Vas (0.7%) had the lowest overall prevalence of malnutrition. The prevalence of overweight and malnutrition in the Han children was 19.2% and 4.2%, respectively (Table 4).

From 1991 to 2010, the Koreans and Mongolians were more likely than the Hans to be obese (Korean: RR, 1.52; 95% CI, 1.48–1.56; Mongol: RR, 1.24; 95% CI, 1.20–1.28). The overall RR for the minority/Han children for overweight was 0.53 (95% CI, 0.52–0.54). Among these minorities, the Dai, Salar, Zhuang, Zang, Dongxiang, and Li were more likely to be malnourished than other minorities. The overall RR for minority/Han children for malnutrition was 0.81 (95% CI, 0.79–0.83) (Table 5).

Further analysis of the trend among the outliers in Fig. 2 showed the prevalence of overweight among the Koreans was obviously increasing and a rising trend was also shown among the Li, Salar, Qiang, and Va children from 1991 to 2010. In the same period, the prevalence of malnutrition was increasing among the Lis, while the prevalence of malnutrition fluctuated slightly among the Korean, Salar, Qiang, and Va children from 1991 to 2010 (Supplementary Tables 6 and 7).

## Discussion

In this nationwide health survey of Chinese children, we observed that the prevalence of overweight in ethnic minorities had increased over the past few decades. Compared with the Hans and excluding Korean and Mongolian children, most ethnic minorities had a lower risk for the prevalence of overweight. The results also indicated that the children living in urban areas had a higher prevalence of overweight than their rural counterparts. Girls had a higher prevalence of malnutrition than boys. Moreover, most minorities had a lower risk for the prevalence of malnutrition among children and adolescents compared with the Hans from 1991 to 2010. Although the overweight prevalence among ethnic minorities was increasing yearly, the trend for malnutrition fluctuated.

Ethnic minorities had a lower prevalence of overweight overall compared with the Hans. A main reason for this may be due to genetic differences between the Hans and some ethnic minorities. For example, the Q27E genotype and allele frequency distribution differs among the Hans and Uyghurs. ADRB2 is associated with fat mobilization and stimulus generated by heat, and its genetic polymorphisms are significantly susceptible to obesity in the human body. Among these polymorphisms, Q27E is most common and is related to diseases such as obesity, primary hypertension, and type 2 diabetes^16^. Furthermore, ethnic groups differ in their dietary habits. Some minorities, such as the Uyghurs and Huis, do not eat pork, owing to their religious beliefs. The Lisu like to eat sour fish and meat made directly from raw materials. The Dais are fond of eating chopped raw beef with seasoning. The Zangs like to eat unseasoned dried beef. Additionally, some minorities, such as the Hanis and Bouyeis, reside in areas with poor transportation, limited communication with the outside world, and in living conditions that are dated and relatively poor. Furthermore, positive physical exercise, which is popular among minorities, could reduce the prevalence of overweight and obesity. For example, the Bamboo Dance is a traditional pastime for the Lis and requires agility to master. The risk of being overweight among the Lis was found to be the lowest.

However, due to the development of local tourism, frequent cultural exchanges, and economic exchanges between the Han and ethnic minorities, the recent prevalence of overweight among ethnic minorities was found to rise. The Mongol and Korean children had a higher overall prevalence of overweight than the Han children, which could be due to different factors. Mongolians living in the plateau region consume a large amount of red meat in order to adapt to the high altitude and frigid weather^17^. Additionally, with accelerated urbanization and economic development in China, socioeconomic status has improved among the Mongolians and Koreans.

Children and adolescents in most ethnic minorities were at a lower risk for malnutrition compared with the Han children from 1991 to 2010. China has adhered to the policy of national equality and unity for an extended period. Furthermore, the Chinese government greatly supports the development of ethnic minorities and has intensified reform and opening-up among minorities. For example, the government gives preferential policies and several subsidies to ethnic minorities. The government also engages in the construction of infrastructures among ethnic minorities, such as hospitals, and trade development, which helps decrease poverty. However, national finance cannot support the entire Han population that lies below the poverty line. Thus, more parents than in the past work far from their hometowns and consequently cannot care for their children. This is a possible reason why the Hans were found to have a relatively high rate of malnutrition compared with ethnic minorities from 1991 to 2010.

The differences among age groups, similar to that of other nations, are more likely caused by children’s growth and sexual maturity than environmental and behavioural factors. Furthermore, we used a statistical definition of obesity and malnutrition that was based on a comparison with the 1985 reference population represented in the CNSSCH growth charts. The charts were created for comparing within specific sex and age groups and not for comparing across sex and age groups. Age differences in prevalence reflect differences from the original reference population. Therefore, we did not focus on the variation from age, and examined the trend of overweight and malnutrition among ethnic minority children, after adjusting for age group as a confounder.

In 1995, the prevalence of malnutrition was high among minorities in the 13–15- and 16–18-year age groups. Mongolian, Shui, Tujia, Bai, Dai, and Yi children were not included in the 1995 survey. The prevalence of malnutrition among the Zang, Uyghur, and Zhuang children in 1995 was higher than that in previous years. Moreover, the Mongolians, Yis, Uyghurs, and Zhuangs had relatively heavier weight. These data may contribute to findings that the prevalence of malnutrition was high among minorities in certain age groups in 1995.

In general, minorities with greater overweight prevalences were less likely to be affected by malnutrition prevalence. Interestingly, results from the Shui, Khalkhas, Lisu, and Monguor children were inconsistent with this finding. The Khalkhas and Monguors are nomadic ethnicities, whereas the Shuis and Lisus livelihoods are based on agriculture, which requires physical strength. This may be the reason why these four ethnicities had a higher prevalence of malnutrition than other minorities.

In China, the overweight prevalence among ethnic minorities increased yearly and the trend of malnutrition fluctuated. However, in developed countries, a stable prevalence of childhood overweight and obesity over the past 15 years has been reported, while the prevalence of malnutrition has been found to be increasing, especially in girls^18^. In a large national survey of Australian school students, the prevalence of overweight and obesity did not increase in the adolescent population overall between 2006 and 2012^19^. In Amsterdam, the prevalence of moderate thinness seems to be increasing^20^. Among 8–15-year-old Bulgarians, an increase in the incidence and prevalence of underweight was observed, with a high percentage of girls aged 14–15 years who were underweight^21^. India is facing similar challenges to China regarding the epidemic trends of overweight and malnutrition. The prevalence of obesity in India has remained somewhat constant over the last couple of decades. However, overweight and combined overweight/obesity prevalence showed an increasing trend^22^. In India, the prevalence of undernutrition remained high despite slowly declining over the past two decades^23^. One study found that one-fifth of Malaysian primary school children were overweight. This finding was comparable to that found in Singapore and Thailand, but was relatively higher than that of Indonesia and Vietnam^24^. However, based on the most recent and representative estimates available in Malaysia, the prevalence of underweight is higher than that of overweight^25^. Moreover, in the urban Pakistan, a four-fold increase in the number of overweight school-aged children in the past 5 years was found, highlighting the alarmingly rapid rise in childhood overweight and obesity^26^. The situation of overweight and malnutrition in neighbouring countries is also serious.

From a public health perspective, it is important to monitor and mitigate trends in overweight and malnutrition among children and adolescents. Non-communicable diseases (NCD) killed 38 million people globally in 2012, accounting for 68% of total annual death worldwide^27^. The increasing trend of obesity, both in adults and children, is directly responsible for the rapid increase in NCDs^28^. Moreover, malnutrition is a risk factor for NCDs related to ageing^29^. Thus, by reducing the prevalence of overweight and malnutrition, the burden of chronic disease can also be reduced, and the life expectancy of humans can be extended.

We acknowledge study limitations. First, this study analysed the Han and 26 other ethnic minority groups, but did not include another 29 ethnic minority groups. However, the remaining ethnic minorities account for <15% of the entire ethnic group population. Second, all Han participants in the 1991 survey were selected from a high socioeconomic population. Therefore, the prevalence of malnutrition is likely underestimated, and the trend of overweight likely overrated. Third, the definitions used for overweight and malnutrition in this article were not global criterions. Although different definitions of overweight and malnutrition in children and adolescents are used worldwide, they may be unsuitable for China’s children and adolescents. However, the standard weight-for-height references were created from the 1985 CNSSCH data (237,476 males and 233,639 females, aged 7–22 years), which had the largest sample size among the six existing surveys. The World Health Organization further recommends using these weight-for-height references. Since 1985, the standard weight-for-height references have been widely used for evaluating children’s nutritional status, and overweight and malnutrition in China.

We observed that the overweight prevalence among ethnic minorities was increasing yearly and that the trend of malnutrition fluctuated. We also examined the relationship between overweight and malnutrition among ethnic minorities. The results of this study could help the Chinese government to better understand the prevalence of overweight and malnutrition among ethnic minorities and take measures to improve the present situation.

## Additional Information

**How to cite this article**: Guo, S. *et al*. Prevalence of overweight and malnutrition among ethnic minority children and adolescents in China, 1991–2010. *Sci. Rep.* **6**, 37491; doi: 10.1038/srep37491 (2016).

**Publisher's note:** Springer Nature remains neutral with regard to jurisdictional claims in published maps and institutional affiliations.

## Supplementary Material

Supplementary Information

## Figures and Tables

**Table 1:** Prevalence of overweight among Chinese minority groups, aged 7–18 years, from 1991 to 2010.

Classification	1991			1995			2000			2005			2010		
	No.	Total	%(95% CI)	No.	Total	%(95% CI)	No.	Total	%(95% CI)	No.	Total	%(95% CI)	No.	Total	%(95% CI)
All	3891	55451	5.8 (5.6–5.9)	3183	54276	4.7 (4.5–4.9)	5774	58551	9.0 (8.7–9.2)	7712	73122	10.4 (10.1–10.6)	10641	80082	13.5 (13.3–13.8)
Sex															
Male	1140	27793	3.4 (3.2–3.6)	1455	27215	4.7 (4.4–4.9)	2646	30264	8.8 (8.5–9.2)	3853	36719	10.8 (10.4–11.1)	5724	40318	15.0 (14.6–15.3)
Female	2751	27658	8.1 (7.8–8.4)	1728	27061	4.7 (4.5–5.0)	3128	28287	9.1 (8.8–9.4)	3859	36403	9.9 (9.6–10.2)	4917	39764	12.0 (11.7–12.4)
Area															
Urban	944	14665	5.1 (4.7–5.4)	671	9555	5.0 (4.6–5.5)	1669	12891	10.6 (10.1–11.2)	1728	11085	11.5 (10.9–12.1)	2427	12503	16.2 (15.6–16.9)
Rural	813	14126	4.7 (4.4–5.1)	515	9591	3.0 (2.7–3.3)	1190	10929	7.4 (6.9–7.9)	1826	14484	9.7 (9.2–10.2)	2280	13651	12.3 (11.8–12.9)
Other	2134	26660	9.0 (8.6–9.3)	1997	35130	6.4 (6.2–6.7)	2915	34731	8.5 (8.2–8.8)	4158	47553	10.0 (9.8–10.3)	5934	53928	12.3 (12.0–12.6)

Note: Other areas is defined as ethnic enclaves where ethnic minorities are living together and cannot be categorized into urban or rural areas.

**Table 2:** Prevalence of malnutrition among Chinese minority groups aged 7–18 years, from 1991 to 2010.

Classification	1991			1995			2000			2005			2010		
	No.	Total	%(95% CI)	No.	Total	%(95% CI)	No.	Total	%(95% CI)	No.	Total	%(95% CI)	No.	Total	%(95% CI)
All	1977	55451	3.6 (3.5–3.8)	2673	54276	6.1 (5.9–6.3)	2616	58551	4.2 (4.1–4.4)	2273	73122	3.3 (3.2–3.5)	3029	80082	4.1 (4.0–4.2)
Sex															
Male	775	27793	3.0 (2.8–3.2)	971	27215	4.4 (4.2–4.7)	758	30264	2.5 (2.3–2.7)	761	36719	2.0 (1.8–2.1)	999	40318	2.7 (2.6–2.9)
Female	1202	27658	4.3 (4.0–4.5)	1702	27061	7.7 (7.4–8.0)	1858	28287	6.1 (5.8–6.4)	1512	36403	4.7 (4.5–4.9)	2030	39764	5.5 (5.3–5.7)
Area															
Urban	575	14665	4.5 (4.1–4.8)	598	9555	7.4 (6.8–7.9)	441	12891	3.8 (3.5–4.2)	352	11085	4.0 (3.6–4.4)	595	12503	5.4 (5.0–5.8)
Rural	351	14126	2.7 (2.4–3.0)	477	9591	5.7 (5.2–6.2)	429	10929	5.0 (4.6–5.4)	343	14484	2.9 (2.6–3.2)	430	13651	3.8 (3.4–4.1)
Other	1051	26660	3.7 (3.5–3.9)	1598	35130	4.9 (4.7–5.1)	1746	34731	4.0 (3.8–4.2)	1578	47553	3.2 (3.0–3.3)	2004	53928	3.3 (3.1–3.5)

Note: The prevalence rates were adjusted according to the minority population based on the sixth nationwide population census (2010).

**Table 3:** Rate ratios for overweight or malnutrition prevalence of minority groups, from 1991 to 2010 [RR (95% CI)].

Parameter	Overweight	Malnutrition
Males/Females	0.91 (0.89–0.93)	0.53 (0.52–0.54)
Survey period	1.24 (1.23–1.25)	0.99 (0.98–0.99)
City		
Other/Urban	0.64 (0.62–0.65)	0.88 (0.86–0.91)
Rural/Urban	0.84 (0.81–0.87)	0.88 (0.87–0.90)
Age group, yr		
10–12/7–9	1.40 (1.36–1.44)	1.79 (1.74–1.85)
13–15/7–9	1.36 (1.32–1.40)	3.30 (3.21–3.39)
16–18/7–9	0.85 (0.82–0.88)	3.25 (3.16–3.34)

**Table 4:** Prevalence of overweight and malnutrition among Chinese minority groups aged 7–18 years in 2010.

Minority	Overweight			Malnutrition		
	Overall	Male	Female	Overall	Male	Female
Bai	9.9 (8.8–11.1)	11.0 (9.3–12.7)	8.7 (7.2–10.3)	6.4 (5.5–7.3)	4.3 (3.2–5.4)	8.8 (7.3–10.4)
Bouyei	9.2 (8.0–10.3)	10.4 (8.7–12.2)	7.7 (6.1–9.2)	2.9 (2.3–3.6)	2.1 (1.3–2.9)	3.9 (2.8–5.0)
Zang or Tibetan	12.4 (11.1–13.6)	14.8 (12.9–16.7)	9.7 (8.1–11.3)	5.8 (4.9–6.7)	4.5 (3.4–5.6)	7.3 (5.9–8.7)
Korean	30.6 (29.4–31.9)	32.8 (31.0–34.6)	28.1 (26.4–29.9)	3.3 (2.8–3.8)	2.0 (1.4–2.5)	4.8 (4.0–5.6)
Dai	6.0 (5.1–6.9)	7.2 (5.8–8.7)	4.6 (3.5–5.8)	8.2 (7.1–9.2)	4.9 (3.7–6.1)	11.8 (10–13.6)
Dongxiang	9.2 (8.0–10.3)	9.9 (8.2–11.6)	8.3 (6.8–9.9)	7.8 (6.7–8.8)	5.4 (4.1–6.7)	10.5 (8.8–12.2)
Dong	10.3 (9.1–11.5)	12.6 (10.7–14.5)	7.7 (6.2–9.2)	4.4 (3.6–5.3)	3.8 (2.7–4.9)	5.1 (3.9–6.4)
Hani	10.0 (8.8–11.1)	9.4 (7.8–11.0)	10.6 (8.9–12.2)	1.8 (1.3–2.3)	1.2 (0.6–1.8)	2.4 (1.6–3.3)
Kazak	18.1 (16.7–19.5)	16.9 (15.0–18.9)	19.4 (17.4–21.5)	1.4 (0.9–1.8)	1.2 (0.6–1.7)	1.6 (0.9–2.2)
Hui	13.9 (13–14.7)	16.3 (15.1–17.5)	10.9 (9.8–12.0)	4.7 (4.2–5.2)	3.2 (2.6–3.8)	6.4 (5.6–7.3)
Khalkhas	7.7 (6.8–8.7)	6.5 (5.2–7.7)	9.2 (7.7–10.7)	2.9 (2.3–3.5)	1.4 (0.8–2.0)	4.6 (3.5–5.7)
Li	3.7 (2.9–4.5)	4.7 (3.5–5.9)	2.6 (1.7–3.5)	10.7(9.4–11.9)	7.5 (6.0–9.0)	14.2 (12.2–16.2)
Lisu	8.0 (6.9–9.0)	6.9 (5.5–8.2)	9.3 (7.7–10.8)	1.5 (1.0–2.0)	1.0 (0.4–1.5)	2.1 (1.3–2.9)
Mongol	22.3 (21.0–23.7)	23.4 (21.5–25.2)	21.2 (19.3–23.0)	1.8 (1.4–2.2)	1.2 (0.7–1.7)	2.5 (1.8–3.2)
Miao	11.6 (10.3–12.9)	11.1 (9.3–12.9)	12.2 (10.3–14.0)	2.3 (1.7–2.9)	2.0 (1.2–2.8)	2.6 (1.7–3.6)
Naxi	13.9 (12.6–15.3)	15.1 (13.2–17.1)	12.5 (10.8–14.3)	3.6 (2.9–4.3)	2.3 (1.5–3.1)	5.0 (3.8–6.2)
Qiang	13.3 (12.1–14.6)	13.2 (11.4–14.9)	13.5 (11.7–15.3)	0.9 (0.6–1.3)	0.3 (0.0–0.6)	1.7 (1.0–2.4)
Salar	8.2 (7.1–9.2)	9.2 (7.6–10.7)	7.0 (5.6–8.4)	10.0 (8.8–11.1)	6.6 (5.2–7.9)	14.0 (12.1–15.9)
Shui	4.7 (3.8–5.5)	5.3 (4.0–6.5)	4.0 (2.9–5.1)	1.9 (1.3–2.4)	1.0 (0.5–1.6)	2.9 (1.9–3.9)
Tujia	19.6 (18.1–21.2)	23.0 (20.7–25.3)	15.8 (13.7–17.8)	1.6 (1.1–2.1)	0.9 (0.4–1.4)	2.4 (1.5–3.2)
Monguor	7.1 (6.1–8.1)	5.5 (4.3–6.8)	8.9 (7.4–10.5)	1.8 (1.3–2.3)	1.0 (0.5–1.6)	2.8 (1.9–3.6)
Uyghur	12.7 (11.9–13.6)	13.9 (12.7–15.2)	11.4 (10.2–12.5)	3.4 (2.9–3.9)	1.9 (1.4–2.4)	5.1 (4.3–5.9)
Yao	14.7 (13.2–16.1)	17.0 (14.8–19.2)	12.0 (10.1–13.9)	2.0 (1.4–2.6)	0.6 (0.1–1.0)	3.6 (2.5–4.7)
Yi	8.3 (7.2–9.3)	7.5 (6.2–8.9)	9.1 (7.6–10.6)	3.6 (2.9–4.3)	3.6 (2.6–4.5)	3.6 (2.6–4.6)
Zhuang	10.1 (9.3–11.0)	11.7 (10.4–12.9)	8.4 (7.3–9.5)	6.8 (6.1–7.5)	4.2 (3.4–5.1)	9.7 (8.5–10.9)
Va	17.9 (16.4–19.3)	16.8 (14.8–18.9)	19.1 (16.9–21.2)	0.7 (0.4–1.0)	0.4 (0.1–0.8)	1.0 (0.5–1.6)
Han	19.2(19.1–19.4)	23.4(23.2–23.7)	14.5(14.2–14.7)	4.2 (4.1–4.3)	2.9 (2.8–3.0)	5.6 (5.5–5.8)

Note: The prevalence rates were adjusted according to the minority population based on the sixth nationwide population census (2010).

**Table 5:** Rate ratios for overweight and malnutrition of minority/Han children, from 1991 to 2010 [RR (95% CI)].

minority	Overweight	Malnutrition
Bai	0.23 (0.21–0.25)	1.06 (0.98–1.16)
Bouyei	0.26 (0.24–0.28)	0.79 (0.72–0.86)
Zang or Tibetan	0.26 (0.25–0.28)	1.42 (1.33–1.52)
Korean	1.52 (1.48–1.56)	0.59 (0.55–0.64)
Dai	0.36 (0.34–0.39)	1.13 (1.04–1.23)
Dongxiang	0.19 (0.17–0.21)	1.45 (1.34–1.58)
Dong	0.27 (0.25–0.29)	0.59 (0.54–0.66)
Hani	0.30 (0.28–0.32)	0.30 (0.26–0.35)
Kazak	0.48 (0.45–0.50)	0.30 (0.25–0.36)
Hui	0.65 (0.63–0.68)	0.91 (0.86–0.97)
Khalkhas	0.29 (0.27–0.31)	0.47 (0.41–0.54)
Li	0.16 (0.15–0.18)	1.47 (1.37–1.57)
Lisu	0.27 (0.25–0.29)	0.32 (0.27–0.39)
Mongol	1.24 (1.20–1.28)	0.41 (0.37–0.45)
Miao	0.42 (0.40–0.44)	0.31 (0.27–0.36)
Naxi	0.33 (0.31–0.36)	0.93 (0.85–1.02)
Qiang	0.30 (0.29–0.32)	0.79 (0.72–0.87)
Salar	0.18 (0.17–0.20)	1.13 (1.04–1.23)
Shui	0.29 (0.27–0.31)	0.38 (0.33–0.44)
Tujia	0.45 (0.42–0.47)	0.42 (0.36–0.49)
Monguor	0.18 (0.16–0.19)	0.89 (0.82–0.98)
Uyghur	0.64 (0.61–0.66)	0.90 (0.85–0.95)
Yao	0.38 (0.36–0.41)	0.52 (0.46–0.59)
Yi	0.20 (0.18–0.23)	0.63 (0.52–0.77)
Zhuang	0.47 (0.45–0.49)	1.35 (1.29–1.42)
Va	0.43 (0.41–0.46)	0.21 (0.17–0.26)
All	0.53 (0.52–0.54)	0.81 (0.79–0.83)

Results adjusted for age, year, sex, and city.
