# Supplementary material for: Prevalence of overweight and malnutrition among ethnic minority children and adolescents in China, 1991–2010
Source: Sci Rep. 2016 Nov 24;6:37491. doi: 10.1038/srep37491 (PMC5121587; doi:10.1038/srep37491)
Supplement: Supplementary Information [file srep37491-s1.docx]

**Prevalence of overweight and malnutrition among ethnic minority children and adolescents in China, 1991–2010**

Sifan Guo^1+^, Chunhua Zhao^1+^, Qinghua Ma^2^, Hongpeng Sun^1*^, Chen-wei Pan^1*^

Supplementary Figure 1. Flow chart of sampling in the 2010 CNSSCH

Chinese Students

Han ethnic

26 ethnic minorities

31 provinces

3 SES groups (upper, middle and lower) per province

48 age groups (7–18 years of age by gender and region) per SES group

Approximately 50 students per age group

13 provinces with ethnic minorities *

48 age groups (7–18 years of age by gender and region) in Mongolian, Hui, Uyghur, Zhuang and Korean minorities;

24 age groups (7–18 years of age by gender) in other ethnic minorities

Approximately 100 students per age group

Note: In step 3, the Hans were chosen from 31 provinces and the 26 ethnic minorities from 13 provinces, as listed below, where the minorities have concentrated settlements.

*Provinces, autonomous regions and municipalities where research activities were undertaken:

Inner Mongolia Province: Mongolian ethnic group; Jilin Province: Korean ethnic group; Hunan: Tujia ethnic group; Hainan Province: Li ethnic group; Guangxi Province: Zhuang and Yao ethnic groups; Sichuan Province: Qiang and Yi ethnic groups; Guizhou Province: Miao, Buyi, and Dong ethnic groups; Yunnan Province: Bai, Hani Dai, Lisu, Wa, and Naxi ethnic groups; Tibet Province: Zang ethnic group; Gansu Province: Dongxiang ethnic group; Qinghai Province: Tu and Salar ethnic groups; Ningxia Province: Hui ethnic group; Sinkiang Province: Uyghur, Kazak, and Khalkhas ethnic groups.

SES: socioeconomic status

Supplementary Table 1. Sample sizes for each year of the CNSSCH

| Minority | 1991 | 1995 | 2000 | 2005 | 2010 |
| --- | --- | --- | --- | --- | --- |
| Bai | 2448 | - | 2401 | 2634 | 2623 |
| Bouyei | 2400 | 2381 | 2380 | 2347 | 2370 |
| Zang or Tibetan | 2377 | 2393 | 2754 | 2373 | 2640 |
| Korean | 4762 | 4797 | 4701 | 5750 | 5241 |
| Dai | 2381 | - | 2336 | 2583 | 2534 |
| Dongxiang | - | 2066 | 2972 | - | 2456 |
| Dong | 2363 | 2400 | 2300 | 2366 | 2391 |
| Hani | 2346 | 2354 | - | 2580 | 2624 |
| Kazak | - | 2383 | - | 2837 | 2900 |
| Hui | 4793 | 4799 | 6191 | 4942 | 6592 |
| Khalkhas | - | 2400 | 1200 | 2867 | 2904 |
| Li | 2399 | 2400 | 2481 | 1865 | 2328 |
| Lisu | - | 2241 | - | 2568 | 2614 |
| Mongol | 6289 | - | 5738 | 4340 | 3834 |
| Miao | 2377 | 2288 | 2179 | 2375 | 2364 |
| Naxi | 2444 | 2400 | - | 2633 | 2633 |
| Qiang | - | 2457 | 2162 | 2759 | 2777 |
| Salar | 1389 | 1199 | 2389 | 2498 | 2607 |
| Shui | 2357 | - | 2157 | 2318 | 2331 |
| Tujia | - | - | 2399 | 2419 | 2517 |
| Monguor | 1379 | 1200 | 2399 | 2560 | 2620 |
| Uyghur | 8148 | 4800 | 2402 | 5735 | 5730 |
| Yao | - | 2400 | 2402 | 2362 | 2306 |
| Yi | - | - | - | - | 2812 |
| Zhuang | 4799 | 4750 | 4788 | 4802 | 4757 |
| Va | - | 2259 | - | 2609 | 2577 |

Supplementary Table 2. Average height (cm) for male Chinese minorities aged 7–18 years, from 1991 to 2010.

| Age | 1991 | 1995 | 2000 | 2005 | 2010 |
| --- | --- | --- | --- | --- | --- |
| 7 | 119.01 | 118.85 | 119.10 | 119.63 | 120.57 |
| 8 | 123.02 | 123.54 | 123.82 | 124.67 | 125.78 |
| 9 | 127.54 | 127.85 | 128.61 | 129.42 | 130.46 |
| 10 | 132.00 | 132.22 | 133.28 | 134.07 | 135.28 |
| 11 | 137.02 | 137.64 | 137.85 | 138.96 | 140.34 |
| 12 | 142.77 | 143.73 | 144.07 | 144.15 | 145.02 |
| 13 | 150.49 | 150.68 | 150.75 | 151.41 | 152.11 |
| 14 | 157.06 | 156.80 | 156.85 | 157.30 | 157.95 |
| 15 | 161.37 | 161.09 | 161.64 | 161.62 | 162.35 |
| 16 | 164.28 | 163.98 | 164.54 | 165.10 | 165.64 |
| 17 | 165.87 | 165.48 | 166.09 | 166.97 | 167.22 |
| 18 | 166.48 | 166.31 | 167.03 | 167.40 | 167.70 |

Supplementary Table 3. Average height (cm) for female Chinese minorities aged 7–18 years, from 1991 to 2010.

| Age | 1991 | 1995 | 2000 | 2005 | 2010 |
| --- | --- | --- | --- | --- | --- |
| 7 | 117.92 | 119.45 | 118.18 | 118.37 | 119.61 |
| 8 | 122.14 | 122.80 | 123.01 | 123.87 | 124.99 |
| 9 | 126.90 | 127.63 | 128.24 | 129.58 | 129.86 |
| 10 | 132.29 | 132.95 | 133.99 | 134.83 | 135.89 |
| 11 | 138.60 | 139.26 | 140.07 | 140.58 | 141.70 |
| 12 | 144.59 | 145.33 | 145.87 | 145.24 | 146.07 |
| 13 | 150.07 | 149.96 | 150.05 | 150.25 | 150.90 |
| 14 | 152.79 | 152.66 | 152.79 | 153.07 | 153.08 |
| 15 | 154.26 | 154.07 | 154.14 | 154.37 | 154.20 |
| 16 | 154.81 | 155.54 | 155.01 | 155.32 | 155.50 |
| 17 | 155.33 | 155.10 | 155.71 | 155.87 | 155.80 |
| 18 | 155.78 | 155.67 | 156.23 | 155.99 | 156.06 |

Supplementary Table 4. Average weight (kg) for male Chinese minorities aged 7–18 years, from 1991 to 2010.

| Age | 1991 | 1995 | 2000 | 2005 | 2010 |
| --- | --- | --- | --- | --- | --- |
| 7 | 20.74 | 21.64 | 21.10 | 22.01 | 22.81 |
| 8 | 22.42 | 22.43 | 23.32 | 24.27 | 25.35 |
| 9 | 24.44 | 24.33 | 25.69 | 26.79 | 27.79 |
| 10 | 27.00 | 26.60 | 28.28 | 29.42 | 30.64 |
| 11 | 30.03 | 29.48 | 30.71 | 32.45 | 33.95 |
| 12 | 33.67 | 33.12 | 34.57 | 35.68 | 36.88 |
| 13 | 39.45 | 38.24 | 39.11 | 41.12 | 42.04 |
| 14 | 44.76 | 42.93 | 44.03 | 45.57 | 46.46 |
| 15 | 48.23 | 47.87 | 48.07 | 49.26 | 50.49 |
| 16 | 51.63 | 49.87 | 51.54 | 53.08 | 53.87 |
| 17 | 53.86 | 51.83 | 53.94 | 55.31 | 56.02 |
| 18 | 54.74 | 53.12 | 55.63 | 56.58 | 57.27 |

Supplementary Table 5 Weight (kg) for female Chinese minorities aged 7–18 years, from 1991 to 2010.

| Age | 1991 | 1995 | 2000 | 2005 | 2010 |
| --- | --- | --- | --- | --- | --- |
| 7 | 19.92 | 19.59 | 20.36 | 20.99 | 21.69 |
| 8 | 21.61 | 21.59 | 22.28 | 23.41 | 24.20 |
| 9 | 23.63 | 23.75 | 24.97 | 26.22 | 26.64 |
| 10 | 26.51 | 26.36 | 27.69 | 29.22 | 29.63 |
| 11 | 30.24 | 30.31 | 31.78 | 32.92 | 34.14 |
| 12 | 34.60 | 34.84 | 35.97 | 36.62 | 37.48 |
| 13 | 40.04 | 39.23 | 40.00 | 41.65 | 42.14 |
| 14 | 43.38 | 42.56 | 43.51 | 45.09 | 45.18 |
| 15 | 45.95 | 45.03 | 46.07 | 47.44 | 47.24 |
| 16 | 47.27 | 43.88 | 47.64 | 49.02 | 49.20 |
| 17 | 48.57 | 47.38 | 49.02 | 49.80 | 49.92 |
| 18 | 48.85 | 47.53 | 49.60 | 50.34 | 50.08 |

Supplementary Table 6. Average overweight (kg) in Korean, Li, Salar, Qiang, Va minorities aged 7–18 years, from 1991 to 2010.

| Minority | 1991 | 1995 | 2000 | 2005 | 2010 |
| --- | --- | --- | --- | --- | --- |
| Korean | 10.09 | 13.57 | 19.38 | 23.36 | 30.63 |
| Li | 2.9 | 1.61 | 5.24 | 7.34 | 3.69 |
| Salar | 1.89 | 1.33 | 4.4 | 5.55 | 8.17 |
| Qiang |  | 5.56 | 6.79 | 9.4 | 13.34 |
| Va |  | 12.94 |  | 9.03 | 17.87 |

Supplementary Table 7. Average overweight (kg) in Korean, Li, Salar, Qiang, Va minorities aged 7–18 years, from 1991 to 2010.

| Minority | 1991 | 1995 | 2000 | 2005 | 2010 |
| --- | --- | --- | --- | --- | --- |
| Korean | 2.17 | 3.19 | 2.9 | 2.3 | 3.27 |
| Li | 4.78 | 8.67 | 6.49 | 9.54 | 10.69 |
| Salar | 4.34 | 8.05 | 6.33 | 7.72 | 9.99 |
| Qiang |  | 2.32 | 2.31 | 1.65 | 0.93 |
| Va |  | 0.8 |  | 1.99 | 0.69 |
